# Genetic Ancestry Estimates within Dutch Family Units and Across Genotyping Arrays: Insights from Empirical Analysis Using Two Estimation Methods

**DOI:** 10.3390/genes14071497

**Published:** 2023-07-22

**Authors:** Jeffrey J. Beck, Talitha Ahmed, Casey T. Finnicum, Koos Zwinderman, Erik A. Ehli, Dorret I. Boomsma, Jouke Jan Hottenga

**Affiliations:** 1 Avera Institute for Human Genetics, Avera McKennan Hospital and University Health Center, Sioux Falls, SD 57105, USA; 2 Department of Biological Psychology, Vrije Universiteit, 1081 HV Amsterdam, The Netherlands; 3 Department of Clinical Epidemiology, Biostatistics, and Bioinformatics, Academic Medical Center Amsterdam, 1105 AZ Amsterdam, The Netherlands; 4 Amsterdam Public Health (APH) Research Institute, 1081 BT Amsterdam, The Netherlands

**Keywords:** within-family analysis, genetic ancestry estimation, population structure, principal components analysis (PCA), ADMIXTURE

## Abstract

Accurate inference of genetic ancestry is crucial for population-based association studies, accounting for population heterogeneity and structure. This study analyzes genome-wide SNP data from the Netherlands Twin Register to compare genetic ancestry estimates. The focus is on the comparison of ancestry estimates between family members and individuals genotyped on multiple arrays (Affymetrix 6.0, Affymetrix Axiom, and Illumina GSA). Two conventional methods, principal component analysis and ADMIXTURE, were implemented to estimate ancestry, each serving its specific purpose, rather than for direct comparison. The results reveal that as the degree of genetic relatedness decreases, the Euclidean distances of genetic ancestry estimates between family members significantly increase (empirical *p* < 0.001), regardless of the estimation method and genotyping array. Ancestry estimates among individuals genotyped on multiple arrays also show statistically significant differences (empirical *p* < 0.001). Additionally, this study investigates the relationship between the ancestry estimates of non-identical twin offspring with ancestrally diverse parents and those with ancestrally similar parents. The results indicate a statistically significant weak correlation between the variation in ancestry estimates among offspring and differences in ancestry estimates among parents (Spearman’s rho: 0.07, *p* = 0.005). This study highlights the utility of current methods in inferring genetic ancestry, emphasizing the importance of reference population composition in determining ancestry estimates.

## 1. Introduction

Genetic association studies have become an effective research tool for identifying genetic loci related to complex phenotypes and diseases [1]. A fundamental step of performing genetic association studies is the detection of and correction for population structure. In this paper, we focus on population structure created by ancestry divergence and its detection based on genotype data. In general, strategies for estimating global ancestry from genetic data can be categorized into two broad groups: algorithmic and model-based approaches. Commonly employed, each method has been shown to provide reliable inferences of genetic ancestry in unrelated individuals and to elucidate population structure from genome-wide data [2].

Algorithmic methods are exemplified by cluster analysis and principal component analysis (PCA). In genetic datasets, PCA is performed to identify systematic variation amongst individuals’ genotypes by a transformation of genotype data into a smaller group of uncorrelated variables, called principal components (PCs), usually with the constraint that each PC successively captures less variation in the original data. PCA of genotypic data yields a series of scores per individual, corresponding to the values of these PCs. Top PCs typically reflect population structure, allowing inferences of genetic ancestry. PCA has demonstrated its utility for elucidating genetic ancestry from seemingly unrelated samples [2], correcting for confounding due to population structure [2,3], and understanding population ancestry composition and migration [4,5,6,7].

The application of PCA in genetic analysis is not without challenges. Care must be taken to ensure that PCs are unbiased and reflect variation in ancestry and not some other form of systematic variation. Rather than capturing population structure, some PCs may reflect linkage disequilibrium (LD) structure. If such PCs are included as covariates in genome-wide association studies (GWASs), the power of these studies is reduced [6,8,9,10]. The degree of population structure captured by PCA may also be diminished by batch effects or family structure. Therefore, commonly employed steps in PCA include determining unrelated individuals, pruning genetic markers in LD, and excluding outlier samples that may be indicative of poor genotyping quality or batch effects.

Model-based approaches, such as those embodied by the programs STRUCTURE [11], fastSTRUCTURE [12], FRAPPE [13], and ADMIXTURE [14], present alternative methods. These approaches provide relative proportions of ancestry and estimate global individual ancestry proportions based on parameterized statistical models. Commonly, these techniques take Bayesian or maximum likelihood estimation approaches to optimize the probability of observed genotypes by alternatively updating ancestry coefficient and population allele frequency matrices. The resulting individual ancestry proportions are thought to be more directly interpretable than PCs, though careful interpretation is warranted [15].

The two methods appear to have little in common at the surface due to underlying analytical differences. One involves the explicit definition of a model, while the other does not. A link between the approaches has been investigated, and strategies for identifying admixture proportions from PCs of PCA have been proposed [16,17,18,19], suggesting ancestry proportions interpreted from PCA and the results of model-based approaches are consistent [20,21]. Given this congruence, the objective of this study was not to directly compare the methods. Instead, the focus was on evaluating ancestry estimates obtained from each method in realistic scenarios where individuals in GWASs possess data from different genotyping arrays due to the utilization of successive generations of genotyping arrays over time.

One strategy for mitigating concerns of population structure in GWASs is to employ a family-based design [22,23,24,25]. With the inclusion of closely related family members new questions may arise. For example, when two individuals from genetically diverse populations mate, their offspring will be admixed, thereby possessing ancestry distributions that differ from both parents. When a child’s ancestry ‘varies’ from its biological parents, the child and at least one parent represent potential population outliers in GWASs, resulting in potential exclusion from the study. Genetic ancestry estimates between the sibling offspring of diverse parents may show increased variation in calculated ancestry due to the random assortment of inherited alleles. We assess the conditions under which such situations may arise by focusing on the sibling offspring of more diverse parents to assess if they are more dissimilar to each other than those with ancestry-similar parents.

In this paper, we examine genetic ancestry estimates between pairs of family members across the spectrum of genetic relatedness. We leverage data from the 1000 Genomes Project (1000 G) [26] and the Genome of the Netherlands (GoNL) [27,28] reference panels as well as multiple large single nucleotide polymorphism (SNP) datasets from participants of the Netherlands Twin Register (NTR) [29,30]. The NTR includes nuclear families, mainly two-generation, forming parent, parent–offspring, dizygotic twin and sibling, and monozygotic twin pairs, all independently genotyped. The NTR also includes SNP datasets of individuals who were genotyped on at least two separate genotyping arrays, allowing for the assessment of genotyping platform effects on genetic ancestry estimates.

## 2. Materials and Methods

An overview of the analytical strategies employed in this study is shown in Figure S1.

### 2.1. Sample Selection and Genotyping

All individuals in the study are participants of the Netherlands Twin Register (NTR) [29,30]. The NTR recruits multiples (i.e., twins and high-order multiples) and their parents, siblings, and spouses to take part in extensive phenotyping and a collection of biological materials for genotyping. DNA from samples provided by the NTR participants was isolated using standard protocols [31]. Individuals were genotyped on Affymetrix 6.0 (Affymetrix Inc., Santa Clara, CA, USA; AFFY6 N_raw individuals_ = 12,779, N_raw variants_ = 905,422), Affymetrix Axiom-NTR [32] (AXIOM N_raw individuals_ = 3606, N_raw variants_ = 642,716), or Illumina GSA-NTR [33] (Illumina Inc., San Diego, CA, USA; ILLGSA N_raw individuals_ = 14,553, N_raw variants_ = 669,322) platforms. Nearly all genotyping was performed at the Avera Institute for Human Genetics (Sioux Falls, SD, USA), with a proportion of those on AFFY6 genotyped at Rutgers University Genomics Center (New Brunswick, NJ, USA). All genotyping was performed according to the respective manufacturer’s protocol.

### 2.2. Dataset Curation

Three platform datasets were created with the backbone and custom content of each array (AFFY6, AXIOM, and ILLGSA). Sample and SNP quality control was conducted on each dataset separately using Plink v1.9 [34]. A Harmonized dataset (61,433 overlapping markers from all three platforms) was created from the cleaned datasets since family members could be genotyped on different arrays. The four datasets underwent the same analytical procedures.

### 2.3. Final Sample Composition

Table 1 describes the final sample after quality control (see Appendix A). Familial relationships were established with Identity-By-Descent (IBD) sharing. In the Harmonized dataset, which enabled family relationships across the genotyping platforms, there were 23,086 unique individuals from 6692 families. There were 3406 MZ twin pairs, 8464 DZ twin or sibling pairs, 16,878 parent–offspring pairs, and 3023 (unrelated) spouse pairs. In each platform dataset, only relationships where family members were genotyped on the same platform were considered. Across all per-platform data, the final sample consisted of 21,117 unique individuals belonging to 6361 families. Of the total per-platform data (three datasets), there were 3258 MZ and 7246 DZ twin or sibling pairs and 13,437 parent–offspring and 2691 spouse pairs.

**Figure 1:**
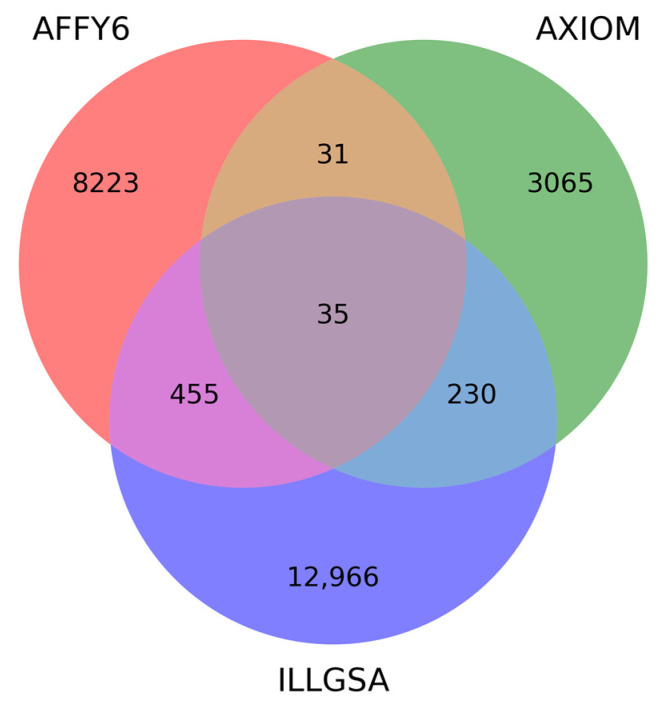
Venn diagram of genotyped NTR individuals according to genotyping platform.

Of the total available sample, 751 individuals were genotyped on at least two of the platforms, 35 of which were genotyped on all three genotyping arrays (Figure 1).

### 2.4. Reference Dataset

Unrelated individuals in the 1000 G (N = 2487) and GoNL (N = 498) reference panels were determined using HapMap3 SNPs. After the alignment of alleles between the reference panels, SNPs present in both datasets were identified (N = 562,607). A final reference set was created with the overlapping markers and subsequent exclusion of SNPs with a call rate of less than 98% (final autosomal marker count: 562,447).

### 2.5. Principal Components Analysis

The largest set of unrelated NTR participants was determined with the KING v2.2.0 software [35] with options --unrelated-degree 2 (i.e., no 1st- or 2nd-degree relationships). In each dataset, unrelated individuals were further filtered to exclude samples with a call rate of less than 95% (a more stringent threshold than the first round of quality control).

The generation and selection of the SNPs for PCA and ADMIXTURE from each of the three platform and Harmonized datasets were determined with the unrelated individual datasets. Autosomal SNPs were selected and filtered to exclude those with a call rate of less than 95%, a minor allele frequency (MAF) of <0.01, and a Hardy–Weinberg Equilibrium (HWE) of *p* < 0.001. SNPs were pruned for linkage disequilibrium (LD) by removing each SNP with an R^2^ value greater than 0.5 with any other SNP within a 250-SNP sliding window (advanced by one SNP each iteration) using Plink v1.9 [34]. Long-range LD regions were removed as previously described [8], resulting in a dataset-specific selection of high-quality, independent SNPs for PCA.

In total, four analysis datasets were generated (three genotyping platforms and the Harmonized set). The selected SNPs of each dataset were then merged with the final reference dataset and filtered to exclude SNPs with a call rate of less than 98% (final SNP count per dataset: AFFY6 = 193,840; AXIOM = 215,848; ILLGSA = 305,121; and Harmonized = 50,030).

For each dataset, 10 PCs were calculated with the SMARTPCA software [16], using 1000 G and GoNL reference populations. PCs were calculated for 27 populations (26 global populations represented in 1000 G plus the GoNL population) and subsequently projected onto all study individuals. PCs were compared between genetic relatedness groups and platforms using descriptive statistics, correlations, and Euclidean distances.

### 2.6. ADMIXTURE Analysis

The cross-validation procedure in ADMIXTURE v.1.3.0 [14,20] was used to identify the value of K, the optimal number of ancestral populations, in the merged 1000 G and GoNL reference panels. The reference data were filtered for a MAF of <0.01 and pruned for LD (SNPs with R^2^ > 0.5 were excluded using a 250 SNP window, advanced by one SNP each iteration). The resulting SNP set (N = 394,918) was analyzed with the cross-validation procedure. The cross-validation method partitions all the observed genotypes into K roughly equally sized folds. The procedure masks all genotypes for each fold in turn. For each fold, the resulting masked dataset is used to calculate estimates of P and Q, the population allele frequencies, and the ancestry proportions, respectively. We varied K from 3 to 27. Prediction errors, reported as the standard error of the cross-validation error estimates, were used to select the model with the lowest error. The optimal model was K = 9, corresponding to the number of distinct ancestral populations (Figure S2).

Study samples were then projected on to the population structure (allele frequencies) of the nine ancestral populations using the learned clusters and ancestry proportions from the K = 9 model of the merged 1000 G and GoNL reference dataset. For each study participant, ancestry proportions of each of the nine demes were calculated (Q1–Q9). The resulting ancestry proportions were compared between genetic relatedness groups and genotyping platforms using descriptive statistics, correlations, and Euclidean distances.

### 2.7. Statistical Analysis

Quantitative evaluation of the genetic ancestry measures was performed on Euclidean distances calculated between pairs of family members based on the relatedness group. For each ancestry estimation method, Euclidean distances were used to quantify differences of the multidimensional data (i.e., 10 PCs or nine ADMIXTURE ancestry proportions) between pairs of individuals with a singular metric. Within each pair of individuals, differences in the PCs or ADMIXTURE ancestry proportions were squared and then summed over all method-specific values. The Euclidean distance was calculated by taking the square root of the summed squared differences (see Formula (1)). In this manner, smaller Euclidean distances represent more similar pairs across all PCs or ADMIXTURE ancestry proportions, whereas larger Euclidean distances indicate a greater dissimilarity across the corresponding values.

Formula (1). Formula for calculating Euclidean distances between pairs of individuals for ten PCs or nine ancestry proportions.

(1) $$d_{x,y}=\sqrt{\sum_{j=1}^{J}{\left(x_{j}-y_{j}\right)}^{2}}$$

*d_x,y_* = Euclidean distance of *J* between two individuals. *x,y* = two individuals, representing a pair within a family. *J* = PCs 1–10 or Q 1–9.

We employed a non-parametric Kruskal–Wallis permutation test (10,000 permutations of relatedness group labels) to assess if there was a difference in ancestry estimates across the four relatedness groups (MZ twins, DZ twins/siblings, parent/offspring, and parent pairs) for each estimation method (PCA and ADMIXTURE). A Kruskal–Wallis test was employed due to the unequal sample sizes of the relatedness groups and the non-normal distributions of the Euclidean distances, particularly in the MZ twin group. A post-hoc Dunn test was used to determine which familial group(s) differed. Using the MZ twin group (genetic control due to being genetically identical), we again implemented the Kruskal–Wallis permutation scheme to evaluate if there were ancestry estimation differences due to the genotyping platform (AFFY6, AXIOM, ILLGSA, and Harmonized) for each estimation method. A post-hoc Dunn test was used to determine which platform(s) differed. We used the same statistical strategy to evaluate differences in ancestry estimates from individuals genotyped on two platforms for both estimation methods. Here, the analysis was based on three groups (individuals with genotypes obtained from AFFY6 and AXIOM, AFFY6 and ILLGSA, or AXIOM and ILLGSA).

## 3. Results

### 3.1. Principal Components Analysis

Table S1 contains descriptive statistics of the PCs. In general, the PCs between platforms are comparable but are not identical since the input SNPs of each dataset varied. Thus, we observed minimal variation in mean values and ranges.

Visualization of the projected PCs 1–10 can be found in Figure S3. To a large extent, the scatter distributions of PCs 1–2 across platforms are nicely superimposed, confirming the similarity of calculated PCs independent of the genotyping platform. Although the same analytical procedures were applied to each dataset, the set of input SNPs for PCA varied. Therefore, the shift of plotted PCs likely reflects differences in input SNPs across genotyping platforms. Shifts are more pronounced in the plots of PC3 vs. PC4 and PC5 vs. PC6. These PCs may be capturing variation attributable to platform-specific SNPs. For the axes showing the most variation, it is plausible that the ILLGSA axes are simply reversed compared to AFFY6 and AXIOM.

To examine the relationship of PCs across datasets representing distinct genotyping arrays, we calculated correlations of PCs 1–10 within and between datasets using the results of array-mimicked reference populations as the input. Correlations of PCs 1–10 within each genotyping platform show no correlation, reflecting the inherent statistical property of PCs in that they are uncorrelated (Figure S4). The correlations of the same PC across platforms are near unity. Negative correlations become apparent for PC3 and PC4 between the AFFY6/AXIOM and ILLGSA platforms. Further divergence of correlations is observed between PCs 6–8, potentially attributable to variation of the platform SNPs.

We next assessed the differences in PCs between the MZ twin and DZ twin/sibling pairs. Since siblings have the same parents, it was expected that the differences in PCs between siblings would be near zero. Since MZ twins arise from the same fertilized egg, the expectation for their PC differences is zero, with non-random values reflecting measurement errors or post-splitting/somatic mutations [36]. The results of comparisons for the MZ twin pairs and DZ twin/sibling pairs are shown in Tables S2 and S3. Mean differences in PCs between the MZ twins were near zero across all ten PCs, irrespective of the genotyping array. The mean differences between the DZ twins/sibling pairs were also near zero across all genotyping platforms. The absolute mean differences in PCs between the MZ twins were less than the DZ twin/sibling pairs across all 10 PCs and genotyping platforms, with few exceptions. The standard deviation of the PC differences in the MZ twins are always smaller than the DZ twins/siblings, reflecting slightly increased variation in PC estimates between non-identical twin siblings.

**Figure 2:**
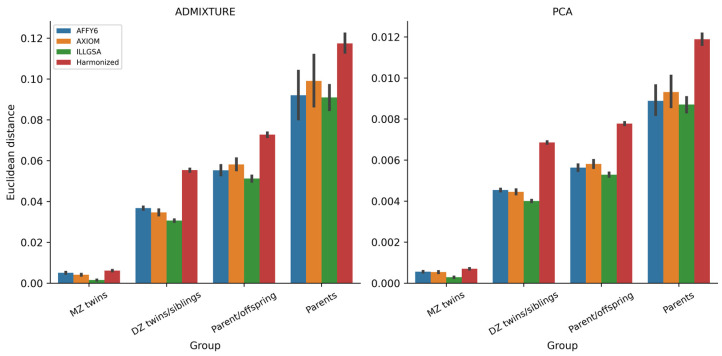
Bar plots of Euclidean distances determined by ADMIXTURE (**left**) and PCA (**right**) by familial group and genotyping array.

According to the relatedness group and dataset, Euclidean distance measures of the PCs are shown in the bar plots in Figure 2 (right panel). There were significant differences in Euclidean distances among all familial groups (empirical *p* < 0.001 and Dunn test *p* < 0.001 for all familial group comparisons). Across all datasets, Euclidean distances were inversely related to the genetic relatedness between the pairs, as expected (Figure S5). That is, highly genetically similar/identical individuals (MZ twin pairs) have smaller Euclidean distances than those that are less genetically similar, such as the DZ twin/sibling pairs. Parent pairs, assumed to be unrelated, have the largest Euclidean distances.

Using the MZ twin group as a genetic control to assess platform effects on genetic ancestry estimates, we observed statistically significant differences across genotyping platforms (empirical *p* < 0.001). Significant differences were observed between platforms produced by different manufacturers (Affymetrix vs. Illumina) and compared to the Harmonized dataset (Dunn test *p* < 0.001 for AFFY6/ILLGSA, AXIOM/ILLGSA, AFFY6/Harmonized, AXIOM/Harmonized, and ILLGSA/Harmonized). There were no significant differences between Euclidean distances in the MZ twins genotyped on AFFY6 and AXIOM (Dunn test *p* = 0.497).

**Figure 3:**
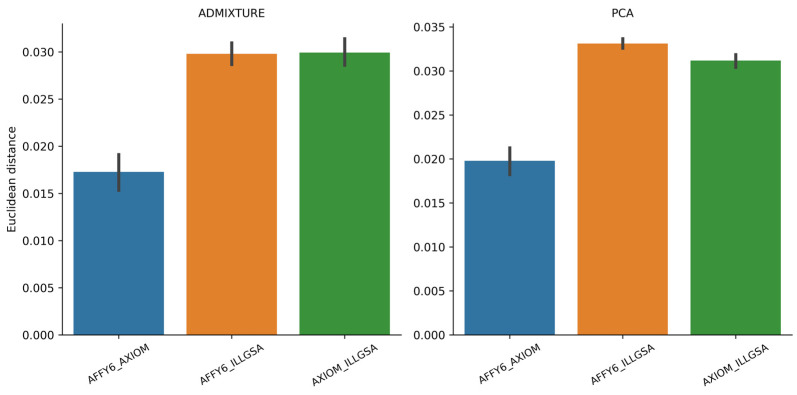
Bar plots of Euclidean distances determined by ADMIXTURE (**left**) and PCA (**right**) for individuals genotyped on multiple platforms.

For individuals with genetic data from multiple genotyping platforms (N = 751; 35 of which were genotyped on all three platforms—see Figure 1), we calculated Euclidean distances of PCs within individuals across the genotyping array (Figure 3, right panel). We expected Euclidean distances near zero, like those between the MZ twins; however, differences of slightly larger magnitude were observed. Statistically significant differences existed between all groups (empirical *p* < 0.001 and Dunn test *p* < 0.001 for all group comparisons). The smallest distance values were obtained for individuals with genotypic data from the AFFY6 and AXIOM platforms. Larger Euclidean distances were observed for individuals with data from each of the array manufacturers: Affymetrix (either AFFY6 or AXIOM) and Illumina (ILLGSA). Because the platform SNPs on which the PCs are based are not identical, the observed differences can be attributed to input SNP variation.

### 3.2. ADMIXTURE Analysis

A global representation of the nine identified ancestral populations (APs) is illustrated in Figure S6. AP1 represents an amalgam of Colombia, Italy, Puerto Rico, and Spain. AP2 predominately reflects Chinese regions (Beijing and Xishuangbanna) and Vietnam. AP3 captures the Finnish population and AP4 represents England, Scotland, and the Netherlands. AP5 and AP6 reflect Peruvian and Mexican populations and Western African populations from Gambia and Sierra Leone, respectively. AP7 symbolizes South Asia, namely Bangladesh, India, Pakistan, and Sri Lanka. AP8 mirrors African populations from Kenya and Nigeria, and AP9 represents East Asian countries, primarily from Japan but also from Beijing, China.

To estimate individual ancestry in the NTR samples, we projected them onto the population structure (allele frequencies) derived from the 1000 G and GoNL reference datasets by specifying K = 9. Descriptive statistics of the ancestral population proportions (Q1–Q9) from the three platform and Harmonized datasets of the NTR participants are shown in Table S4. Across datasets, there was little variation in mean ancestry proportions. AP4/Q4 represents the majority of the NTR individuals, indicating that most of the genetic ancestry corresponds to the 1000 G and GONL reference data obtained from Northern and Western Europeans and the Netherlands.

The stacked bar charts in Figure S7A–D display the ancestry proportion estimates of each NTR individual per genotyping platform and in the Harmonized dataset. Each stacked bar reflects a single individual and their ancestry fractions for the nine populations, arranged in increasing order of AP4. The average Q of AP4 is 0.695, 0.687, 0.687, and 0.694 from AFFY6, AXIOM, ILLGSA, and Harmonized data, respectively. Across all data, there is a modest amount of ancestry captured by AP1 with average Q estimates of 0.187, 0.191, 0.191, and 0.188.

As observed in the stacked bar charts, the ancestral fractions of all the NTR individuals are nearly indistinguishable from each other for across platforms. This finding highlights a relatively similar population composition of individuals genotyped on each platform. Comparatively, PCs can reveal more fine-grained differences between the same individuals, such as North–South or East–West clines. Within each platform dataset, a small number of genetically diverse and admixed individuals are shown on the left side of each figure. These individuals show stark variation in the ancestry proportions relative to the bulk of the NTR sample population, indicating more heterogenous ancestry and deviation from the majority Northern and Western Europe origin as captured by AP4. Similar admixture and population heterogeneity patterns among the NTR samples were observed in the PCs (Figure S3F–J).

Correlations of ancestry proportions within and between genotyping platforms for all the NTR participants are shown in Figure S8. Within each ancestral population (Q1–Q9), the estimates are strongly correlated between the genotyping arrays. For values of Q within the genotyping platforms, ancestry estimates are mostly negatively correlated or not correlated at all. Between values of Q and between the genotyping platforms, estimates are also mainly negatively correlated or not correlated at all. Exceptions include positive correlations between Q2 and Q7 as well as Q2 and Q9, reflecting a moderate overlap in the South and East Asian populations. There were also slightly positive correlations between Q5 and Q8, and Q6 and Q8 obtained from the AXIOM and ILLGSA arrays. The moderately strong correlation between the Q6 and Q8 correlation is likely due to the overlap of the African populations comprising each ancestral population.

We compared the estimates between the MZ and between DZ twins/sibling pairs to examine the ancestry proportions in more detail. The results are shown in Tables S5 and S6. Mean differences between the MZ twins were near zero across all ancestry proportions and genotyping arrays. The same was true for the DZ twins/siblings, but the mean differences are nearly always smaller between the MZ twins than between the DZ twins/siblings.

Consistent with the evaluation of PCs, we calculated Euclidean distances over the nine ancestry proportions within family pairs according to Formula (1). Comparable to the Euclidean distances of PCs, the distances in ancestry proportions increased as the degree of relatedness between the pairs of individuals became less (Figure 2, left panel). There was a statistically significant difference between all familial groups (empirical *p* < 0.001 and Dunn test *p* < 0.001 for all familial group comparisons).

Again, using the MZ twins as a genetic control group, we compared Euclidean distance measures as a function of the genotyping platform within this group. Overall, we observed statistically significant differences (empirical *p* < 0.001). The individual platform comparisons all yielded statistically significant differences (Dunn test *p* < 0.001), except for the comparison between the AFFY6 and Harmonized platforms.

We also investigated the ancestry proportions of individuals genotyped on multiple platforms. Except for the AFFY6_ILLGSA and AXIOM_ILLGSA group comparison (Dunn test *p* = 0.404), statistically significant differences existed for all group comparisons (empirical *p* < 0.001 and Dunn test *p* < 0.001). Like the Euclidean distances of PCs of individuals genotyped on multiple arrays, the smallest distances were observed for those with genetic data obtained from the Affymetrix platforms (Figure 3, left panel). Larger distances were observed when individuals were genotyped on the Affymetrix and Illumina platforms.

### 3.3. Ancestry Outliers—PCA vs. ADMIXTURE

Ancestry outliers were identified by defining thresholds based on minimum and maximum PC and ancestry proportion values of CEU or GoNL reference populations. For PCA, thresholds were defined for each platform and the Harmonized set. PCs from the CEU and GoNL individuals were calculated with datasets mimicking the content of each platform or the Harmonized dataset. CEU and GoNL platform-specific thresholds were not possible for ADMIXTURE since the nine populations were determined with an LD-pruned dataset of markers present in both 1000 G and GoNL panels. Each NTR dataset was projected onto the reference populations.

Ancestry outliers were defined as having PCs or ADMIXTURE proportions less than or greater than reference (i.e., CEU or GoNL) minima or maxima, respectively. The NTR individuals with values greater than or equal to the reference minimum or less than or equal to the reference maximum were considered inliers. Outliers were determined for each PC and each value of Q. The total number of outliers across all PCs and values of Q was determined by identifying unique individuals.

Table 2 shows the number of outliers and inliers per dataset with thresholds determined by the CEU or GoNL reference populations. The number of outliers between the PCA and ADMIXTURE are very similar when thresholds were defined by the larger GoNL reference population (N = 498). A larger deviation in outlier counts is observed for CEU (N = 99)-defined boundaries, which is a smaller and more ancestrally variable population than GoNL. Regardless of the reference population, there is more variation in outlier counts in the Harmonized dataset, likely due to the smaller number of markers used in the calculations.

### 3.4. Assessment of Within-Family Diversity

**Figure 4:**
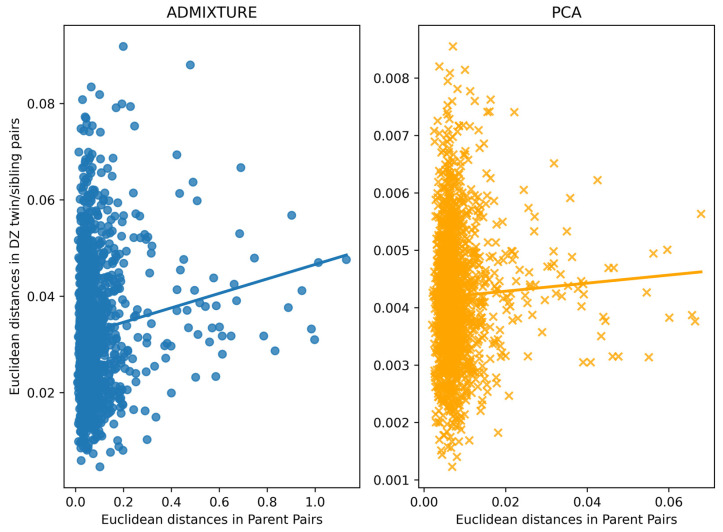
Scatter plot of Euclidean distances determined by ADMIXTURE (**left**) and PCA (**right**) between parent pairs (*x*-axis) and DZ twin/sibling pairs averaged within a family (*y*-axis). A regression line is shown for each plot.

Using the calculated PCs and ADMIXTURE ancestry proportions, we also assessed if the sibling offspring (non-MZ) of diverse parents were more dissimilar to each other than those with parents of similar ancestry (Figure 4). Euclidean distances of the DZ twin and sibling offspring were averaged within a family to avoid inflating the number of comparisons in families with multiple offspring. We found small positive correlations between Euclidean distances of parent pairs (i.e., father and mother) and averaged distances of all DZ twin and sibling pairs within a family (ADMIXTURE Spearman’s rho 0.07, *p* = 0.005; PCA Spearman’s rho 0.04, *p* = 0.122). Overall, the Euclidean distances calculated from the PCs were smaller in magnitude than those derived from ADMIXTURE ancestry proportions. Though the ADMIXTURE comparison yielded a statistically significant correlation, it was a very weak positive relationship. Thus, the sibling offspring of more diverse parents show slightly more variation in ancestry to each other compared to the sibling offspring with parents of similar genetic ancestry. However, this weak relationship appears to depend on the method used for calculating ancestry estimates. Regardless of the method, the near-zero relationship indicates that the sibling offspring of more diverse parents are slightly more dissimilar than the progeny of parents of similar genetic ancestry.

## 4. Discussion

We examined ancestry estimates as a function of the genotyping array and genetic relatedness within nuclear families and evaluated estimates of genetic ancestry obtained from PCA and ADMIXTURE in twin pairs and their family members based on whole-genome SNP data from three genotyping platforms and a harmonized dataset. Utilizing reference data from 1000 G and GoNL as global population surrogates, we demonstrated that PCs across genotyping arrays are not the same despite identical analytical strategies. We ascribe this finding to differences in platform SNPs. Across familial groups, the Euclidean distance measures of PCs were inversely related to the degree of genetic similarity between individuals. The greater the genetic relatedness between two individuals, the smaller the Euclidean distances of their respective PCs. Given that the twin/sibling offspring have the same parents and possess a genetic profile derived from the same pool of segregating alleles, this finding is in line with theoretical expectations. However, statistically significant differences were observed between all familial groups, even between the DZ twin/siblings and parent/offspring pairs, which would be expected to be ~50% similar. These differences might also be explained by the fact that within pairs of a familial group, there are variable numbers of ancestry outliers. This intrinsic difference would result in greater variation in the Euclidean distance measures.

We used ADMIXTURE, a model-based ancestry estimation method, to detect the optimal number of ancestral populations in the global reference data. Ancestral population 4 was the major ancestry fraction of the NTR participants, representing Northern and Western Europe and the Netherlands. The differences in ancestry proportions of the nine populations were near zero between the MZ twins and DZ twins/siblings. Consistent with the evaluation of PCs, the Euclidean distance measures of ancestry proportions were inversely proportional to the amount of allele sharing between family members.

Given the uniqueness of the NTR data, we evaluated estimates of genetic ancestry from genotypic data from the independently genotyped MZ twins. Importantly, within-platform differences of the MZ twins were non-zero. Although measurement error may play a role, differences within the MZ twin pairs cannot simply be ascribed to measurement error alone. One DNA sequencing study showed in a 40- and a 100-year-old MZ twin pair that somatic mosaicism leads to differences within pairs [37], and more recently, germline differences were shown in a large Icelandic study of the genomes in pedigrees of MZ twins [36]. The relative contribution of germline differences and measurement errors as sources of variation in ancestry estimates for MZ twins remains to be determined.

We examined ancestry estimates of individuals genotyped on multiple arrays and observed the largest Euclidean distances between the Affymetrix (AFFY6/AXIOM) and Illumina (ILLGSA) platforms. These variations can be attributed to array manufacturer differences, including array design, chemistry, and platform-specific genotype calling algorithms. The bar plots of the Euclidean distances for the MZ twins, who should be genetically identical, also exhibit these differences. It is crucial to consider the unequal sample sizes per platform, the variability in the composition of genotyped individuals, and the limited number of samples with genotypes from multiple arrays, which impose limitations on the study’s findings. Regardless of the ancestry estimation method and familial group, the Euclidean distances for individuals genotyped on the ILLGSA platform are smaller than the other platform datasets. This finding is consistent with the fact that the Illumina GSA-NTR array includes an improved backbone for capturing population variation and was further customized to include additional content to aid in population-specific GWASs [33]. Perhaps the inclusion of these additional population-relevant markers enhances the input into PCA and ADMIXTURE, in turn providing better population resolution at the level of the individual. This platform enhancement reflects the small(est) observed differences in ancestry estimates in MZ twins, which would be expected to be essentially zero.

The accuracy of ancestry inference methods depends on various factors, including the distribution of human genetic variation across geographic regions, the types and number of genetic markers used, the sampled population, the choice of reference populations, and the statistical methods for interpreting variation patterns [38]. Ancestry exists on a continuum due to the complexity of human evolution and migrations [39]. In this study, the spectrum of ancestry considered is limited to the diversity represented in the global surrogate samples from the 1000 G and GoNL projects. As more extensive and diverse genetic datasets become available [40,41], finer resolution estimates of genetic ancestry will be achievable.

When investigating genetic ancestry, a variety of statistical tests have been recommended for selecting the number of PCs (e.g., Tracy–Widom statistics [16]) or ancestral populations from ADMIXTURE (e.g., Bayesian information criterion [20]). Meanwhile, others advise that these decisions be made based on the knowledge of the history of the study population(s) [21] or additional investigative analysis [7]. The top 10 PCs of the PCA method are often included in association studies for adjusting for the population structure [42,43,44,45], which is the number of PCs we considered in this project. It is possible that further examination of ancestry estimates derived from PCA, will lead to utilizing additional PCs. Future studies examining ancestry estimates from genotype data that are coordinated and aggregated via imputation would also be of merit.

For many years, PCA has been widely used in genetic association studies to address population stratification confounding. However, recent scrutiny has raised concerns about potential biases associated with this technique in population genetic research [46] and GWASs [47]. In this study, we employed a projection-based PCA approach, and our results highlight the utility of this method. Importantly, our PCA findings demonstrate consistency with the results obtained from ADMIXTURE and align well with theoretical expectations, particularly when evaluating genetic ancestry within family members. While acknowledging the potential pitfalls associated with PCA in population genetic studies [7], our research emphasizes the relevance of the projection-based PCA approach and provides valuable insights into the estimation of genetic ancestry in familial contexts.

Overall, we show genetic ancestry inference methods can provide reliable estimates of individual genetic ancestry across the genetic relatedness spectrum from genetic data from various genotyping arrays. The consistency of the estimates is contingent upon the inclusion of necessary proxies of global population diversity and proper analytical execution. Genetic relatedness can confound individual ancestry estimates in the absence of appropriate reference population samples [45]. Several methods for handling relatedness in PCA have been proposed [48,49] that rely on performing PCA on diverse unrelated individuals first with subsequent PC prediction based on genetic similarities. To mitigate the concern of genetic relatedness, we utilized projection strategies to select independent SNPs for PCA and ADMIXTURE analyses based on unrelated individuals from globally diverse reference populations. We demonstrated that PCs and ancestry proportions from ADMIXTURE show minor differences between closely related pairs of individuals (i.e., MZ twins) and that these differences are not consistent between genotyping platforms. Though platform differences were apparent, relatively consistent results were observed from PCA and ADMIXTURE. From a population genetics perspective, ancestry proportion estimates may be more favorable than PCA since they are more easily interpretable. That is because ADMIXTURE returns proportions of membership to surrogate global ancestral populations, whereas PCA simply reveals axes of variation in the data. Regardless of the preferred method, the performance of PCA and ADMIXTURE for estimating genetic ancestry is comparable for downstream analyses including family members genotyped on multiple platforms.

## Figures and Tables

**Table 1:** Final NTR sample description after quality control and filtering.

Genotyping Platform	Unique Families	Unique Individuals	MZ Twin Pairs	DZ Twin/Sibling Pairs	Parent–Offspring Pairs	Parent Pairs
AFFY6	2800	7575	1279	2966	2849	438
AXIOM	734	2593	433	591	2222	448
ILLGSA	3562	11,597	1546	3689	8366	1805
Across all platforms	6361	21,177	3528	7246	13,437	2691
Harmonized	6692	23,086	3406	8464	16,878	3023

**Table 2:** Outliers and inliers from PCA and ADMIXTURE based on two reference datasets.

	Min. and Max. Thresholds Determined by GoNL ^1^					Min. and Max. Thresholds Determined by CEU ^2^				
	PCA ^3^		ADMIXTURE ^4^		Common Outliers ^5^	PCA ^3^		ADMIXTURE ^4^		Common Outliers ^5^
Dataset (N)	Outliers (%)	Inliers (%)	Outliers (%)	Inliers (%)		Outliers (%)	Inliers (%)	Outliers (%)	Inliers (%)	
AFFY6 (8744)	525 (6.0%)	8219 (94.0%)	695 (7.9%)	8049 (92.1%)	457	818 (9.4%)	7926 (90.6%)	2419 (27.7%)	6325 (72.3%)	704
AXIOM (3361)	249 (7.4%)	3112 (92.6%)	341 (10.1%)	3020 (89.9%)	224	451 (13.4%)	2910 (86.6%)	896 (26.7%)	2465 (73.3%)	382
ILLGSA (13,686)	933 (6.8%)	12,753 (93.2%)	954 (7.0%)	12,732 (93.0%)	809	1169 (8.5%)	12,517 (91.5%)	2501 (18.3%)	11,185 (81.7%)	891
Harmonized (25,005)	1794 (7.2%)	23,211 (92.8%)	5617 (22.5%)	19,388 (77.5%)	1584	3438 (13.7%)	21,567 (86.3%)	12,501 (50.0%)	12,504 (50.0%)	2796

^1^ Sample size is 498. ^2^ Sample size is 99. ^3^ Min. and max. thresholds determined within each dataset (498 GONL and 99 CEU reference samples mimicking the content of each dataset). ^4^ Min. and max. thresholds determined from entire reference dataset (GONL and CEU) and not within each dataset. ^5^ Number of outliers is shared between each method.

## Data Availability

Data are available on request due to privacy restrictions. The data presented in this study are available on request from the Netherland’s Twin Register data access committee, which reviews data requests and makes data available to interested researchers. The data are not publicly available because they come from extended twin families and pedigrees, which creates privacy concerns.

## Supplementary Materials

The following supporting information can be downloaded at: https://www.mdpi.com/article/10.3390/genes14071497/s1, Figure S1: Process flowchart of the analytical strategies employed in this study; Figure S2: Results of the cross-validation procedure determined by ADMIXTURE using the 1000 G and GoNL reference datasets; Figure S3: Scatterplots of PCs 1–10 across genotyping platforms; Figure S4: Correlations of PCs across genotyping array; Figure S5: Scatterplot of the proportion of IBD (PIHAT) and Euclidean distance between pairs by platform; Figure S6: World view of the nine populations as determined by ADMIXTURE; Figure S7: Results of ADMIXTURE analysis for each genotyping array with K = 9. Figure S8: Correlations of ancestry proportions across genotyping array. Table S1: Descriptive statistics of PCs of NTR participants; Table S2: Within-family MZ twin pair differences in PCs by genotyping array; Table S3: Within-family DZ twin/sibling pair differences in PCs by genotyping array; Table S4: Descriptive statistics of ADMIXTURE ancestry proportions of NTR participants; Table S5: Within-family MZ twin differences in ADMIXTURE ancestry proportions by genotyping array; Table S6: Within-family DZ twin/sibling pair differences in ADMIXTURE ancestry proportions by genotyping array.

## Code Availability Statement

Scripts for the PCA and ADMIXTURE analysis can be found at https://github.com/jjbeck821/genes_genetic_ancestry (accessed on 19 July 2023).

## Appendix A

Details of sample and SNP quality control procedures.

Samples were excluded if phenotypic sex did not match the genotypic sex (indicating potential sample swap) (N = 271; 0.88%), if the Plink heterozygosity F value was <−0.10 or >0.10 (N = 292; 0.94%), or if the sample call rate was less than 90% (N = 16; 0.05%). Within families, pairwise identity-by-descent (IBD) was estimated with Plink v1.9 [34], and samples were removed if they did not match the expected familial relations (indicating potential sample swap) (N = 312; 1.00%). NTR samples present in GoNL or related to GoNL participants were excluded (N = 51; 0.16%).

Next, SNPs were evaluated in each platform and were excluded if they fit any of the following criteria: minor allele frequency (MAF) < 0.005, HWE p-value < 0.00001, SNP call rate <95%, or Mendelian error rate >2%. SNPs were also removed if they were palindromic, A/T or C/G alleles, with an allele frequency between 0.40–0.60. All platform data were aligned to build 37 of the Human Genome (hg19), and alleles were flipped to the plus strand if needed. SNPs were also removed if the allele frequencies differed more than 0.10 with the GoNL reference panel (AFFY6 N = 81 of 640265 [0.01%], AXIOM N = 52 of 589258 [0.01%], GSA N = 233 of 497095 [0.05%]).

## References

[1] Visscher P.M., Wray N.R., Zhang Q., Sklar P., McCarthy M.I., Brown M.A., Yang J. (2017). 10 Years of GWAS Discovery: Biology, Function, and Translation. Am. J. Hum. Genet..

[2] Price A.L., Patterson N.J., Plenge R.M., Weinblatt M.E., Shadick N.A., Reich D. (2006). Principal components analysis corrects for stratification in genome-wide association studies. Nat. Genet..

[3] Novembre J., Stephens M. (2008). Interpreting principal component analyses of spatial population genetic variation. Nat. Genet..

[4] Novembre J., Johnson T., Bryc K., Kutalik Z., Boyko A.R., Auton A., Indap A., King K.S., Bergmann S., Nelson M.R. (2008). Genes mirror geography within Europe. Nature.

[5] Reich D., Price A.L., Patterson N. (2008). Principal component analysis of genetic data. Nat. Genet..

[6] Abdellaoui A., Hottenga J.-J., de Knijff P., Nivard M.G., Xiao X., Scheet P., Brooks A., Ehli E.A., Hu Y., Davies G.E. (2013). Population structure, migration, and diversifying selection in the Netherlands. Eur. J. Hum. Genet..

[7] Prive F., Luu K., Blum M.G.B., McGrath J.J., Vilhjalmsson B.J. (2020). Efficient toolkit implementing best practices for principal component analysis of population genetic data. Bioinformatics.

[8] Price A.L., Weale M.E., Patterson N., Myers S.R., Need A.C., Shianna K.V., Ge D., Rotter J.I., Torres E., Taylor K.D. (2008). Long-range LD can confound genome scans in admixed populations. Am. J. Hum. Genet..

[9] Zou F., Lee S., Knowles M.R., Wright F.A. (2010). Quantification of population structure using correlated SNPs by shrinkage principal components. Hum. Hered..

[10] Prive F., Aschard H., Ziyatdinov A., Blum M.G.B. (2018). Efficient analysis of large-scale genome-wide data with two R packages: Bigstatsr and bigsnpr. Bioinformatics.

[11] Pritchard J.K., Stephens M., Rosenberg N.A., Donnelly P. (2000). Association mapping in structured populations. Am. J. Hum. Genet..

[12] Raj A., Stephens M., Pritchard J.K. (2014). fastSTRUCTURE: Variational inference of population structure in large SNP data sets. Genetics.

[13] Tang H., Peng J., Wang P., Risch N.J. (2005). Estimation of individual admixture: Analytical and study design considerations. Genet. Epidemiol..

[14] Alexander D.H., Lange K. (2011). Enhancements to the ADMIXTURE algorithm for individual ancestry estimation. BMC Bioinform..

[15] Lawson D.J., van Dorp L., Falush D. (2018). A tutorial on how not to over-interpret STRUCTURE and ADMIXTURE bar plots. Nat. Commun..

[16] Patterson N., Price A.L., Reich D. (2006). Population structure and eigenanalysis. PLoS Genet..

[17] Engelhardt B.E., Stephens M. (2010). Analysis of population structure: A unifying framework and novel methods based on sparse factor analysis. PLoS Genet..

[18] McVean G. (2009). A genealogical interpretation of principal components analysis. PLoS Genet..

[19] Ma J., Amos C.I. (2012). Principal components analysis of population admixture. PLoS ONE.

[20] Alexander D.H., Novembre J., Lange K. (2009). Fast model-based estimation of ancestry in unrelated individuals. Genome Res..

[21] Zheng X., Weir B.S. (2016). Eigenanalysis of SNP data with an identity by descent interpretation. Popul. Biol..

[22] Abecasis G.R., Cardon L.R., Cookson W.O. (2000). A general test of association for quantitative traits in nuclear families. Am. J. Hum. Genet..

[23] Benyamin B., Visscher P.M., McRae A.F. (2009). Family-based genome-wide association studies. Pharmacogenomics.

[24] Brumpton B., Sanderson E., Heilbron K., Hartwig F.P., Harrison S., Vie G.A., Cho Y., Howe L.D., Hughes A., Boomsma D.I. (2020). Avoiding dynastic, assortative mating, and population stratification biases in Mendelian randomization through within-family analyses. Nat. Commun..

[25] Howe L.J., Nivard M.G., Morris T.T., Hansen A.F., Rasheed H., Cho Y., Chittoor G., Ahlskog R., Lind P.A., Palviainen T. (2022). Within-sibship genome-wide association analyses decrease bias in estimates of direct genetic effects. Nat. Genet..

[26] Genomes Project C., Auton A., Brooks L.D., Durbin R.M., Garrison E.P., Kang H.M., Korbel J.O., Marchini J.L., McCarthy S., McVean G.A. (2015). A global reference for human genetic variation. Nature.

[27] Boomsma D.I., Wijmenga C., Slagboom E.P., Swertz M.A., Karssen L.C., Abdellaoui A., Ye K., Guryev V., Vermaat M., van Dijk F. (2014). The Genome of the Netherlands: Design, and project goals. Eur. J. Hum. Genet..

[28] Genome of the Netherlands C. (2014). Whole-genome sequence variation, population structure and demographic history of the Dutch population. Nat. Genet..

[29] Willemsen G., Vink J.M., Abdellaoui A., den Braber A., van Beek J.H., Draisma H.H., van Dongen J., van ’t Ent D., Geels L.M., van Lien R. (2013). The Adult Netherlands Twin Register: Twenty-five years of survey and biological data collection. Twin Res. Hum. Genet..

[30] van Beijsterveldt C.E., Groen-Blokhuis M., Hottenga J.J., Franic S., Hudziak J.J., Lamb D., Huppertz C., de Zeeuw E., Nivard M., Schutte N. (2013). The Young Netherlands Twin Register (YNTR): Longitudinal twin and family studies in over 70,000 children. Twin Res. Hum. Genet..

[31] Min J.L., Lakenberg N., Bakker-Verweij M., Suchiman E., Boomsma D.I., Slagboom P.E., Meulenbelt I. (2006). High microsatellite and SNP genotyping success rates established in a large number of genomic DNA samples extracted from mouth swabs and genotypes. Twin Res. Hum. Genet..

[32] Ehli E.A., Abdellaoui A., Fedko I.O., Grieser C., Nohzadeh-Malakshah S., Willemsen G., de Geus E.J., Boomsma D.I., Davies G.E., Hottenga J.J. (2017). A method to customize population-specific arrays for genome-wide association testing. Eur. J. Hum. Genet..

[33] Beck J.J., Hottenga J.J., Mbarek H., Finnicum C.T., Ehli E.A., Hur Y.M., Martin N.G., de Geus E.J.C., Boomsma D.I., Davies G.E. (2019). Genetic Similarity Assessment of Twin-Family Populations by Custom-Designed Genotyping Array. Twin Res. Hum. Genet..

[34] Chang C.C., Chow C.C., Tellier L.C., Vattikuti S., Purcell S.M., Lee J.J. (2015). Second-generation PLINK: Rising to the challenge of larger and richer datasets. Gigascience.

[35] Manichaikul A., Mychaleckyj J.C., Rich S.S., Daly K., Sale M., Chen W.M. (2010). Robust relationship inference in genome-wide association studies. Bioinformatics.

[36] Jonsson H., Magnusdottir E., Eggertsson H.P., Stefansson O.A., Arnadottir G.A., Eiriksson O., Zink F., Helgason E.A., Jonsdottir I., Gylfason A. (2021). Differences between germline genomes of monozygotic twins. Nat. Genet..

[37] Ouwens K.G., Jansen R., Tolhuis B., Slagboom P.E., Penninx B., Boomsma D.I. (2018). A characterization of postzygotic mutations identified in monozygotic twins. Hum. Mutat..

[38] Royal C.D., Novembre J., Fullerton S.M., Goldstein D.B., Long J.C., Bamshad M.J., Clark A.G. (2010). Inferring genetic ancestry: Opportunities, challenges, and implications. Am. J. Hum. Genet..

[39] Akey J.M., Eberle M.A., Rieder M.J., Carlson C.S., Shriver M.D., Nickerson D.A., Kruglyak L. (2004). Population history and natural selection shape patterns of genetic variation in 132 genes. PLoS Biol..

[40] Lemke A.A., Esplin E.D., Goldenberg A.J., Gonzaga-Jauregui C., Hanchard N.A., Harris-Wai J., Ideozu J.E., Isasi R., Landstrom A.P., Prince A.E. (2022). Addressing underrepresentation in genomics research through community engagement. Am. J. Hum. Genet..

[41] Sirugo G., Williams S.M., Tishkoff S.A. (2019). The Missing Diversity in Human Genetic Studies. Cell.

[42] Price A.L., Zaitlen N.A., Reich D., Patterson N. (2010). New approaches to population stratification in genome-wide association studies. Nat. Rev. Genet..

[43] Kang S.J., Larkin E.K., Song Y., Barnholtz-Sloan J., Baechle D., Feng T., Zhu X. (2009). Assessing the impact of global versus local ancestry in association studies. BMC Proc..

[44] Feng Q., Abraham J., Feng T., Song Y., Elston R.C., Zhu X. (2009). A method to correct for population structure using a segregation model. BMC Proc..

[45] Thornton T., Conomos M.P., Sverdlov S., Blue E.M., Cheung C.Y., Glazner C.G., Lewis S.M., Wijsman E.M. (2014). Estimating and adjusting for ancestry admixture in statistical methods for relatedness inference, heritability estimation, and association testing. BMC Proc..

[46] Elhaik E. (2022). Principal Component Analyses (PCA)-based findings in population genetic studies are highly biased and must be reevaluated. Sci. Rep..

[47] Akond Z., Ahsan M.A., Alam M., Mollah M.N.H. (2021). Robustification of GWAS to explore effective SNPs addressing the challenges of hidden population stratification and polygenic effects. Sci. Rep..

[48] Zhu X., Li S., Cooper R.S., Elston R.C. (2008). A unified association analysis approach for family and unrelated samples correcting for stratification. Am. J. Hum. Genet..

[49] Conomos M.P., Miller M.B., Thornton T.A. (2015). Robust inference of population structure for ancestry prediction and correction of stratification in the presence of relatedness. Genet. Epidemiol..

