# Supplementary material for: Genetic Ancestry Estimates within Dutch Family Units and Across Genotyping Arrays: Insights from Empirical Analysis Using Two Estimation Methods
Source: Genes (Basel). 2023 Jul 22;14(7):1497. doi: 10.3390/genes14071497 (PMC10379078; doi:10.3390/genes14071497)
Supplement: Supplementary file 1 [file genes-14-01497-s001.zip › Supplementary_Tables/pdfs/Table_S1.pdf]

Supplementary Table 1 – Descriptive statistics of principal components of NTR participants

	AFFY6					AXIOM					ILLGSA					HARMONIZED				
	Mean	SD	Min	Max	Range	Mean	SD	Min	Max	Range	Mean	SD	Min	Max	Range	Mean	SD	Min	Max	Range
PC1	0.0112	0.0015	-0.0338	0.0121	0.0459	0.0114	0.0022	-0.0313	0.0124	0.0437	0.0143	0.0022	-0.0352	0.0156	0.0508	0.0116	0.0019	-0.0359	0.0130	0.0489
PC2	0.0176	0.0037	-0.0344	0.0194	0.0538	0.0174	0.0038	-0.0342	0.0191	0.0533	0.0159	0.0037	-0.0365	0.0176	0.0541	0.0172	0.0037	-0.0368	0.0196	0.0564
PC3	-0.0070	0.0023	-0.0196	0.0395	0.0591	-0.0069	0.0029	-0.0191	0.0385	0.0576	0.0070	0.0023	-0.0422	0.0197	0.0619	-0.0068	0.0025	-0.0240	0.0431	0.0671
PC4	-0.0087	0.0015	-0.0161	0.0380	0.0541	-0.0085	0.0020	-0.0147	0.0446	0.0593	0.0083	0.0017	-0.0500	0.0164	0.0664	-0.0089	0.0019	-0.0176	0.0475	0.0651
PC5	0.0020	0.0033	-0.0403	0.0284	0.0687	0.0059	0.0056	-0.0442	0.0266	0.0708	0.0061	0.0053	-0.0478	0.0501	0.0979	0.0004	0.0033	-0.0469	0.0423	0.0892
PC6	0.0069	0.0049	-0.0452	0.0327	0.0779	0.0035	0.0036	-0.0226	0.0401	0.0627	-0.0083	0.0041	-0.0350	0.0288	0.0638	0.0069	0.0057	-0.0525	0.0481	0.1006
PC7	-0.0002	0.0014	-0.0055	0.0052	0.0107	0.0002	0.0014	-0.0095	0.0222	0.0317	-0.0073	0.0041	-0.0289	0.0345	0.0634	-0.0005	0.0024	-0.0310	0.0197	0.0507
PC8	0.0049	0.0023	-0.0131	0.0134	0.0265	0.0113	0.0050	-0.0251	0.0231	0.0482	-0.0002	0.0013	-0.0179	0.0332	0.0511	0.0035	0.0030	-0.0308	0.0163	0.0471
PC9	0.0112	0.0045	-0.0206	0.0303	0.0509	-0.0038	0.0022	-0.0112	0.0087	0.0199	0.0003	0.0025	-0.0181	0.0190	0.0371	0.0112	0.0054	-0.0309	0.0315	0.0624
PC10	0.0004	0.0023	-0.0085	0.0152	0.0237	0.0005	0.0022	-0.0123	0.0091	0.0214	-0.0006	0.0017	-0.0307	0.0109	0.0416	-0.0006	0.0034	-0.0174	0.0180	0.0354

PC1-PC10=principal components 1 through 10, SD=standard deviation, Min=minimum, Max=maximum
