# Supplementary material for: Genetic Ancestry Estimates within Dutch Family Units and Across Genotyping Arrays: Insights from Empirical Analysis Using Two Estimation Methods
Source: Genes (Basel). 2023 Jul 22;14(7):1497. doi: 10.3390/genes14071497 (PMC10379078; doi:10.3390/genes14071497)
Supplement: Supplementary file 1 [file genes-14-01497-s001.zip › Supplementary_Tables/pdfs/Table_S2.pdf]

Supplementary Table 2 - Within family MZ twin pair differences in PCs by genotyping array

	AFFY6 (N=1279)				AXIOM (N=433)				ILLGSA (N=1546)				Harmonized (N=3406)			
	MD	SD	MAD	IQRAD	MD	SD	MAD	IQRAD	MD	SD	MAD	IQRAD	MD	SD	MAD	IQRAD
PC1	0.0000020	0.0001606	0.0001	0.0001	0.0000014	0.0001309	0	0.0001	-0.0000002	0.0001520	0	0.0001	0.0000042	0.0001432	0	0.0001
PC2	0.0000013	0.0001771	0.0001	0.0001	0.0000012	0.0001436	0.0001	0.0001	-0.0000039	0.0001722	0	0.0001	0.0000022	0.0001929	0	0.0001
PC3	-0.0000038	0.0001050	0.0001	0.0001	-0.0000025	0.0000973	0.0001	0.0001	-0.0000018	0.0000819	0	0.0001	-0.0000041	0.0001296	0.0001	0.0001
PC4	0.0000000	0.0001178	0.0001	0.0001	0	0.0000962	0.0001	0.0001	0.0000007	0.0001031	0	0.0001	-0.0000041	0.0001392	0.0001	0.0001
PC5	-0.0000085	0.0001717	0.0001	0.0002	0.0000187	0.0002288	0.0001	0.0001	-0.0000017	0.0001378	0	0.0001	-0.0000001	0.0002559	0.0001	0.0002
PC6	-0.0000010	0.0002334	0.0001	0.0001	0.0000058	0.0001777	0.0001	0.0002	0.0000026	0.0001419	0	0.0001	0.0000102	0.0003171	0.0001	0.0002
PC7	0.0000034	0.0002173	0.0001	0.0001	0.0000115	0.0002006	0.0001	0.0001	0.0000026	0.0001357	0.0001	0.0001	-0.0000076	0.0002859	0.0001	0.0002
PC8	0.0000099	0.0002456	0.0001	0.0001	0.0000088	0.0002843	0.0001	0.0002	-0.0000010	0.0001044	0	0.0001	0.0000104	0.0003477	0.0001	0.0002
PC9	-0.0000005	0.0002663	0.0001	0.0002	0.0000229	0.0002111	0.0001	0.0001	0.0000033	0.0001463	0.0001	0.0001	0.0000027	0.0003933	0.0002	0.0003
PC10	0.0000024	0.0002565	0.0001	0.0002	0.0000136	0.0002727	0.0002	0.0002	0.0000025	0.0001324	0	0.0001	0.0000046	0.0003907	0.0002	0.0003

MD and SD are the mean and standard deviation of paired principal component differences, MAD = median absolute difference, IQRAD = interquartile range absolute difference of quartile 1 – quartile 3
