# Supplementary material for: Genetic Ancestry Estimates within Dutch Family Units and Across Genotyping Arrays: Insights from Empirical Analysis Using Two Estimation Methods
Source: Genes (Basel). 2023 Jul 22;14(7):1497. doi: 10.3390/genes14071497 (PMC10379078; doi:10.3390/genes14071497)
Supplement: Supplementary file 1 [file genes-14-01497-s001.zip › Supplementary_Tables/pdfs/Table_S3.pdf]

Supplementary Table 3 – Within family DZ twin/sibling pair differences in PCs by genotyping array

	AFFY6 (N=2966)				AXIOM (N=591)				ILLGSA (N=3689)				Harmonized (N=8464)			
	MD	SD	MAD	IQRAD	MD	SD	MAD	IQRAD	MD	SD	MAD	IQRAD	MD	SD	MAD	IQRAD
PC1	-0.0000027	0.0002658	0.0002	0.0002	-0.0000249	0.0002404	0.0002	0.0001	0.0000043	0.0002733	0.0001	0.0001	-0.0000006	0.0003408	0.0002	0.0003
PC2	-0.0000179	0.0004050	0.0002	0.0003	-0.0000218	0.0003827	0.0002	0.0003	0.0000124	0.0003816	0.0002	0.0002	0.0000050	0.0005172	0.0003	0.0004
PC3	0.0000049	0.0006581	0.0004	0.0006	0.0000538	0.0006119	0.0004	0.0005	0.0000076	0.0005788	0.0004	0.0005	0.0000129	0.0009118	0.0006	0.0008
PC4	0.0000281	0.0006870	0.0005	0.0006	-0.0000294	0.0006448	0.0004	0.0006	0.0000062	0.0006056	0.0004	0.0005	0.0000008	0.0009624	0.0006	0.0008
PC5	-0.0000189	0.0015276	0.0010	0.0012	0.0000088	0.0019236	0.0014	0.0017	0.0000002	0.0017806	0.0012	0.0014	-0.0000367	0.0022051	0.0015	0.0018
PC6	-0.0000097	0.0020585	0.0014	0.0017	0.0001037	0.0015581	0.0010	0.0013	0.0000539	0.0016400	0.0011	0.0014	-0.0000418	0.0028650	0.0020	0.0024
PC7	-0.0000297	0.0013230	0.0009	0.0010	0.0000091	0.0013700	0.0009	0.0011	0.0000183	0.0016578	0.0011	0.0014	0.0000016	0.0023673	0.0016	0.0019
PC8	0.0000152	0.0017449	0.0012	0.0015	-0.0000959	0.0022387	0.0016	0.0020	0.0000259	0.0012354	0.0008	0.0010	-0.0000485	0.0027616	0.0019	0.0023
PC9	-0.0000432	0.0022698	0.0015	0.0019	-0.0001530	0.0015666	0.0011	0.0014	-0.0000784	0.0020788	0.0014	0.0018	-0.0000309	0.0033817	0.0022	0.0027
PC10	-0.0000735	0.0021903	0.0015	0.0018	-0.0000922	0.0022229	0.0016	0.0018	-0.0000031	0.0014639	0.0010	0.0013	-0.0000133	0.0033483	0.0022	0.0027

MD and SD are the mean and standard deviation of paired principal component differences, MAD = median absolute difference, IQRAD = interquartile range absolute difference of quartile 1 – quartile 3.
