# Supplementary material for: Genetic Ancestry Estimates within Dutch Family Units and Across Genotyping Arrays: Insights from Empirical Analysis Using Two Estimation Methods
Source: Genes (Basel). 2023 Jul 22;14(7):1497. doi: 10.3390/genes14071497 (PMC10379078; doi:10.3390/genes14071497)
Supplement: Supplementary file 1 [file genes-14-01497-s001.zip › Supplementary_Tables/pdfs/Table_S4.pdf]

Supplementary Table 4 – Descriptive statistics of admixture proportions of NTR participants

	AFFY6					AXIOM					ILLGSA					HARMONIZED				
	Mean	SD	Min	Max	Range	Mean	SD	Min	Max	Range	Mean	SD	Min	Max	Range	Mean	SD	Min	Max	Range
Q1	0.1869	0.0587	0.0000	0.7468	0.7468	0.1908	0.0663	0.0039	0.7405	0.7366	0.1948	0.0596	0.0000	0.7194	0.7194	0.1878	0.0658	0.0000	0.7608	0.7608
Q2	0.0112	0.0527	0.0000	0.8731	0.8731	0.0114	0.0538	0.0000	0.8164	0.8164	0.0106	0.0505	0.0000	0.8937	0.8937	0.0113	0.0511	0.0000	0.8807	0.8807
Q3	0.0725	0.0220	0.0000	0.4332	0.4332	0.0726	0.0212	0.0000	0.3204	0.3204	0.0756	0.0206	0.0000	0.6184	0.6184	0.0702	0.0312	0.0000	0.6213	0.6213
Q4	0.6949	0.0919	0.0000	0.9314	0.9314	0.6868	0.1041	0.0000	0.8588	0.8588	0.6833	0.0954	0.0000	0.9218	0.9218	0.6936	0.1024	0.0000	0.9575	0.9575
Q5	0.0054	0.0100	0.0000	0.4484	0.4484	0.0054	0.0138	0.0000	0.5075	0.5074	0.0055	0.0117	0.0000	0.5525	0.5525	0.0060	0.0126	0.0000	0.5576	0.5575
Q6	0.0036	0.0154	0.0000	0.4681	0.4681	0.0049	0.0216	0.0000	0.4586	0.4585	0.0041	0.0186	0.0000	0.5581	0.5581	0.0043	0.0185	0.0000	0.5584	0.5584
Q7	0.0171	0.0419	0.0000	0.8840	0.8840	0.0189	0.0530	0.0000	0.8491	0.8491	0.0174	0.0400	0.0000	0.9001	0.9001	0.0173	0.0442	0.0000	0.9330	0.9330
Q8	0.0039	0.0167	0.0000	0.5172	0.5171	0.0049	0.0229	0.0000	0.5952	0.5952	0.0043	0.0197	0.0000	0.6412	0.6412	0.0044	0.0199	0.0000	0.6672	0.6672
Q9	0.0045	0.0144	0.0000	0.5465	0.5465	0.0042	0.0123	0.0000	0.3438	0.3438	0.0043	0.0205	0.0000	0.7515	0.7515	0.0050	0.0183	0.0000	0.7733	0.7733

Q1-Q9 represent each of the nine ancestry populations as determined by ADMIXTURE, SD=standard deviation, Min=minimum, Max=maximum
