# Supplementary material for: Genetic Ancestry Estimates within Dutch Family Units and Across Genotyping Arrays: Insights from Empirical Analysis Using Two Estimation Methods
Source: Genes (Basel). 2023 Jul 22;14(7):1497. doi: 10.3390/genes14071497 (PMC10379078; doi:10.3390/genes14071497)
Supplement: Supplementary file 1 [file genes-14-01497-s001.zip › Supplementary_Tables/pdfs/Table_S5.pdf]

Supplementary Table 5 – Within family MZ twin differences in ancestry proportions by genotyping array

	AFFY6 (N=1279)				AXIOM (N=433)				ILLGSA (N=1546)				Harmonized (N=3406)			
	MD	SD	MAD	IQRAD	MD	SD	MAD	IQRAD	MD	SD	MAD	IQRAD	MD	SD	MAD	IQRAD
Q1	-0.0000237	0.0022813	0.0013	0.0018	-0.0002526	0.0022389	0.0011	0.0016	0.0000071	0.0009782	0.0004	0.0005	-0.0000953	0.0038340	0.0015	0.0028
Q2	0.0000270	0.0012185	0.0002	0.0009	-0.0000023	0.0008463	0.0002	0.0007	-0.0000115	0.0005284	0.0001	0.0003	0.0000143	0.0014961	0.0000	0.0007
Q3	-0.0000005	0.0020525	0.0012	0.0016	-0.0000082	0.0020162	0.0010	0.0015	0.0000181	0.0009788	0.0003	0.0006	0.0000385	0.0035242	0.0013	0.0024
Q4	0.0000402	0.0049464	0.0018	0.0027	0.0003037	0.0033462	0.0017	0.0025	-0.0000141	0.0013523	0.0005	0.0007	0.0001686	0.0055211	0.0020	0.0038
Q5	0.0000148	0.0007813	0.0003	0.0006	-0.0000164	0.0005789	0.0002	0.0005	-0.0000032	0.0003113	0.0001	0.0002	-0.0000189	0.0010212	0.0002	0.0007
Q6	0.0000550	0.0015490	0.0003	0.0010	-0.0000545	0.0009789	0.0002	0.0006	0.0000016	0.0004610	0	0.0002	-0.0000358	0.0013595	0.0000	0.0004
Q7	-0.0000285	0.0016026	0.0007	0.0012	-0.0000099	0.0012470	0.0006	0.001	-0.0000011	0.0006435	0.0002	0.0003	-0.0000667	0.0021005	0.0005	0.0015
Q8	-0.0000545	0.0021503	0.0003	0.0010	0.0000075	0.0011565	0.0001	0.0006	-0.0000215	0.0004775	0	0.0002	-0.0000230	0.0014435	0.0000	0.0005
Q9	-0.0000298	0.0010583	0.0001	0.0008	0.0000326	0.0007626	0	0.0005	0.0000245	0.0004302	0	0.0002	0.0000183	0.0014032	0.0000	0.0006

Q1-Q9 represent each of the nine ancestry populations as determined by ADMIXTURE, MD and SD are the mean and standard deviation of paired ancestry proportion differences, MAD = median absolute difference, IQRAD = interquartile range absolute difference of quartile 1 – quartile 3.
