# Supplementary material for: Genetic Ancestry Estimates within Dutch Family Units and Across Genotyping Arrays: Insights from Empirical Analysis Using Two Estimation Methods
Source: Genes (Basel). 2023 Jul 22;14(7):1497. doi: 10.3390/genes14071497 (PMC10379078; doi:10.3390/genes14071497)
Supplement: Supplementary file 1 [file genes-14-01497-s001.zip › Supplementary_Tables/pdfs/Table_S6.pdf]

Supplementary Table 6 – Within family DZ twin/sibling pair differences in admixture proportions by genotyping array

	AFFY6 (N=2966)				AXIOM (N=591)				ILLGSA (N=3689)				Harmonized (N=8464)			
	MD	SD	MAD	IQRAD	MD	SD	MAD	IQRAD	MD	SD	MAD	IQRAD	MD	SD	MAD	IQRAD
Q1	0.0001091	0.0218368	0.0144	0.0184	-0.0010909	0.0208740	0.0144	0.0176	0.0003525	0.0183653	0.0126	0.0154	0.0007276	0.0324349	0.0219	0.0269
Q2	0.0000416	0.0066079	0.0023	0.0056	0.0004513	0.0056273	0.0023	0.0054	-0.0000435	0.0061190	0.0025	0.0051	-0.0001435	0.0088039	0.0022	0.0077
Q3	0.0000445	0.0169808	0.0115	0.0144	0.0002999	0.0164323	0.0115	0.0127	0.0002883	0.0141443	0.0094	0.012	-0.0002675	0.0279527	0.0189	0.0237
Q4	-0.0005116	0.0255068	0.0173	0.0212	0.0004375	0.0233512	0.0173	0.0202	-0.0005890	0.0202821	0.0138	0.0166	-0.0005575	0.0386821	0.0258	0.0322
Q5	0.0001855	0.0044754	0.0028	0.0039	-0.0000242	0.0042301	0.0028	0.0039	0.0001141	0.0040468	0.0024	0.0034	0.0000168	0.0065354	0.0034	0.0066
Q6	-0.0000331	0.0041066	0.0020	0.0042	0.0000620	0.0037017	0.0020	0.0039	-0.0000086	0.0036473	0.0017	0.0038	0.0000588	0.0054829	0.0013	0.0053
Q7	0.0001313	0.0094434	0.0061	0.0081	-0.0001375	0.0087924	0.0061	0.0081	-0.0002554	0.0083539	0.0055	0.007	0.0002028	0.0130998	0.0075	0.0123
Q8	0.0000234	0.0040089	0.0018	0.0042	0.0003426	0.0042484	0.0018	0.0043	0.0001301	0.0040864	0.0019	0.0038	-0.0000872	0.0056071	0.0015	0.0053
Q9	0.0000092	0.0052608	0.0020	0.0054	-0.0003406	0.0053752	0.0020	0.0053	0.0000114	0.0050228	0.0018	0.0048	0.0000496	0.0075707	0.0015	0.0069

Q1-Q9 represent each of the nine ancestry populations as determined by ADMIXTURE, MD and SD are the mean and standard deviation of paired ancestry proportion differences, MAD = median absolute difference, IQRAD = interquartile range absolute difference of quartile 1 – quartile 3.
