# Supplementary figures and images for: Genetic Ancestry Estimates within Dutch Family Units and Across Genotyping Arrays: Insights from Empirical Analysis Using Two Estimation Methods
Source: Genes (Basel). 2023 Jul 22;14(7):1497. doi: 10.3390/genes14071497 (PMC10379078; doi:10.3390/genes14071497)

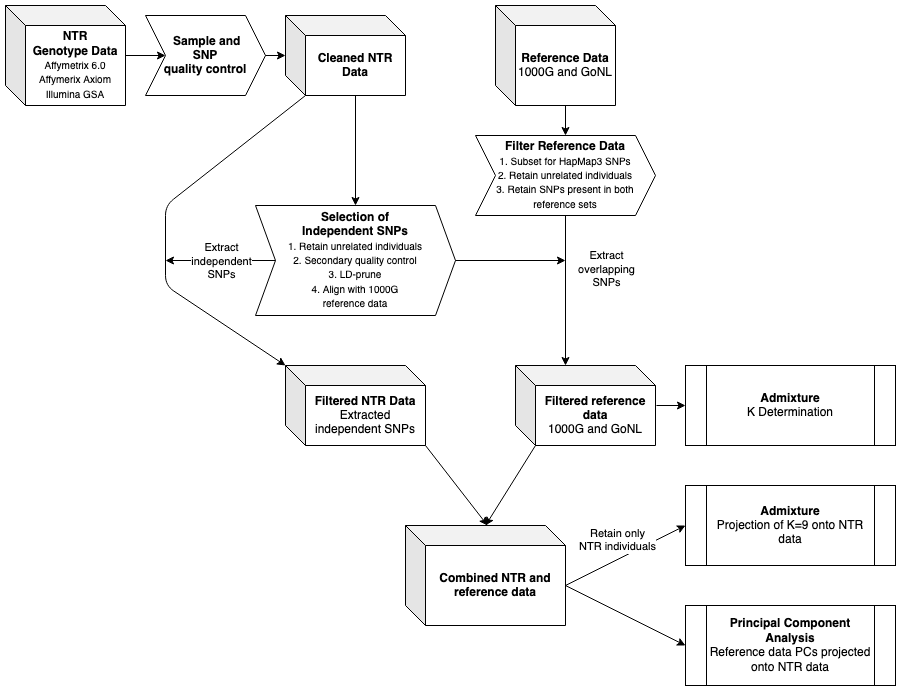

Supplement: Supplementary file 1 [file genes-14-01497-s001.zip › Supplementary_Figures/Figure_S1.png]

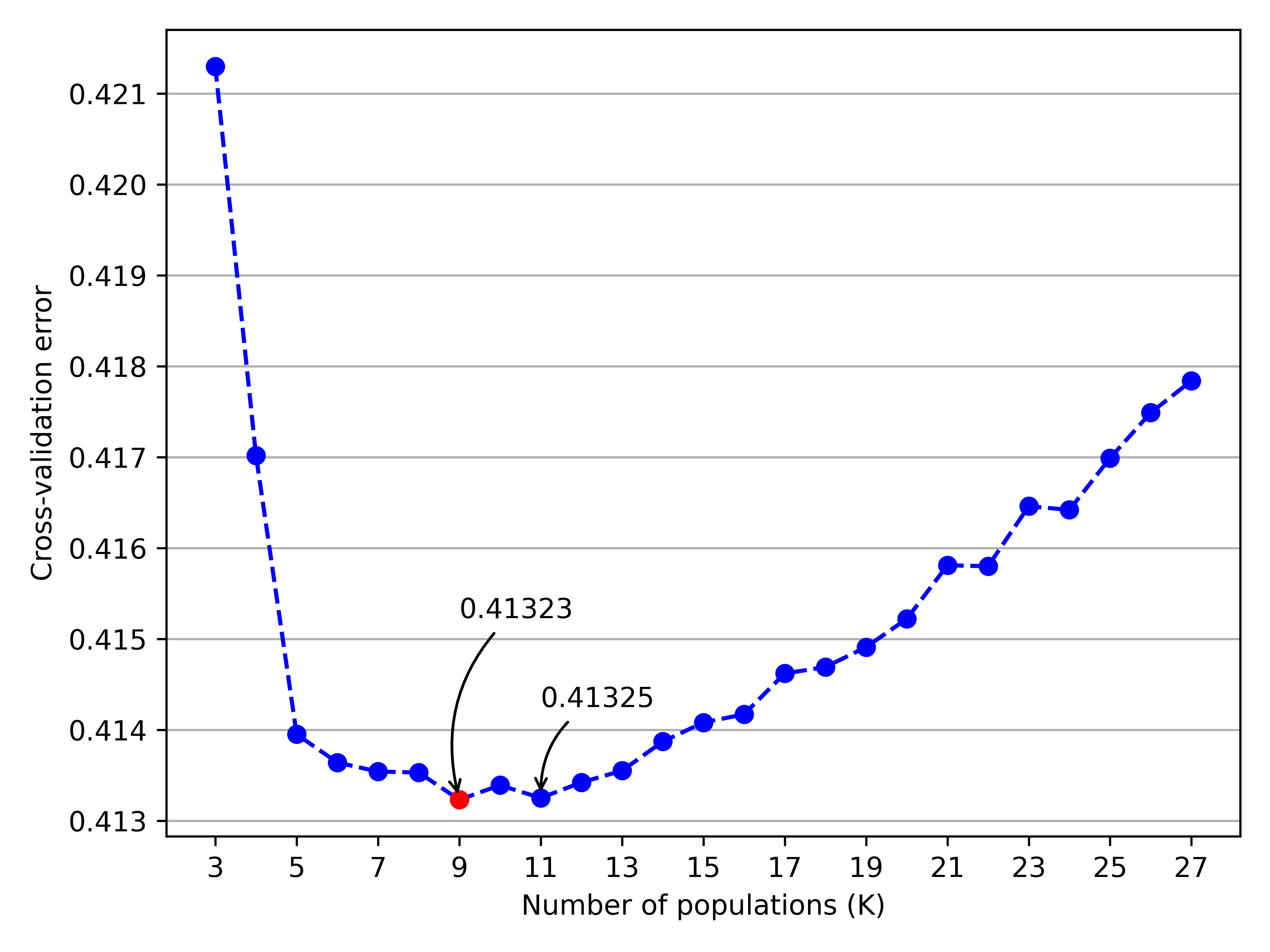

Supplement: Supplementary file 1 [file genes-14-01497-s001.zip › Supplementary_Figures/Figure_S2.png]

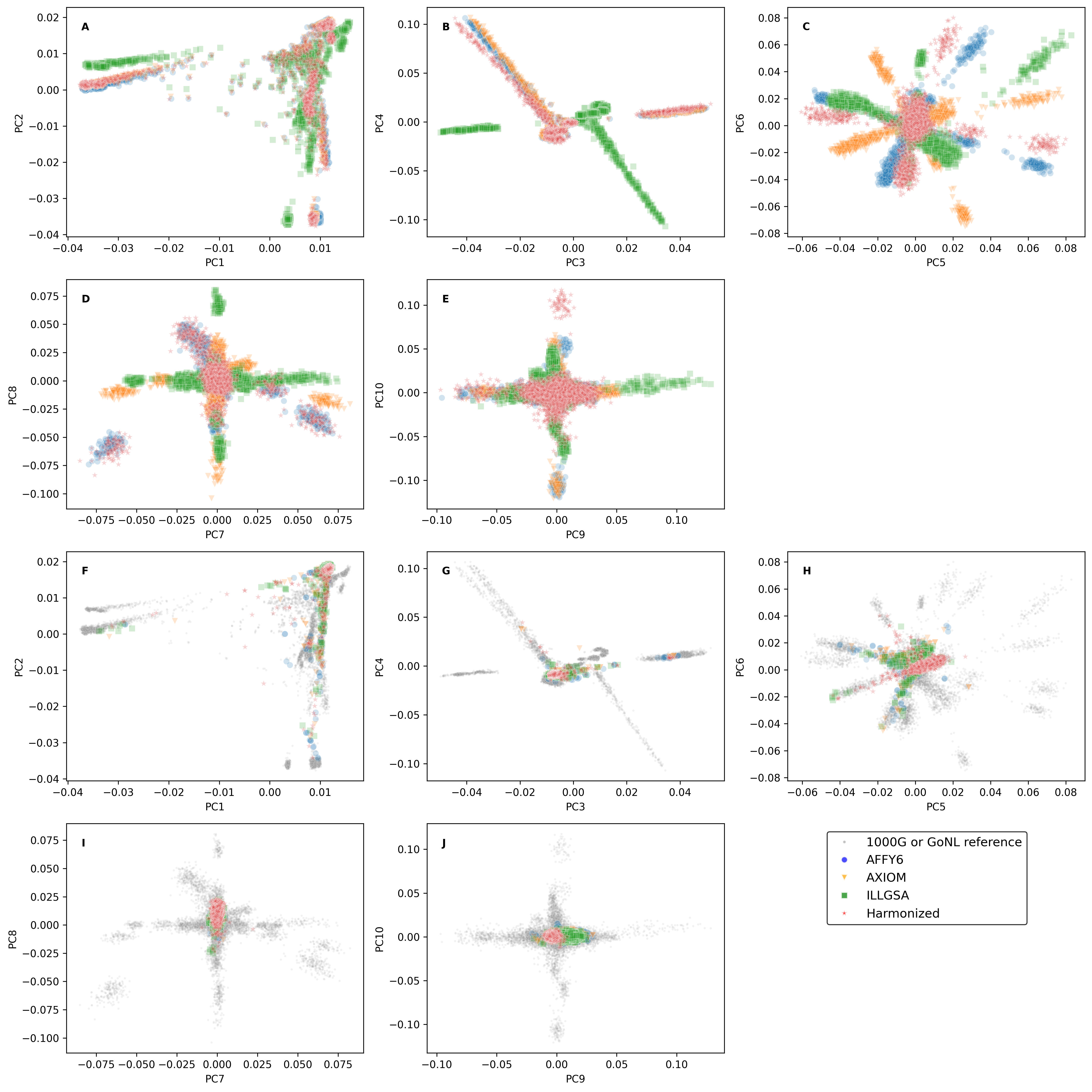

Supplement: Supplementary file 1 [file genes-14-01497-s001.zip › Supplementary_Figures/Figure_S3.png]

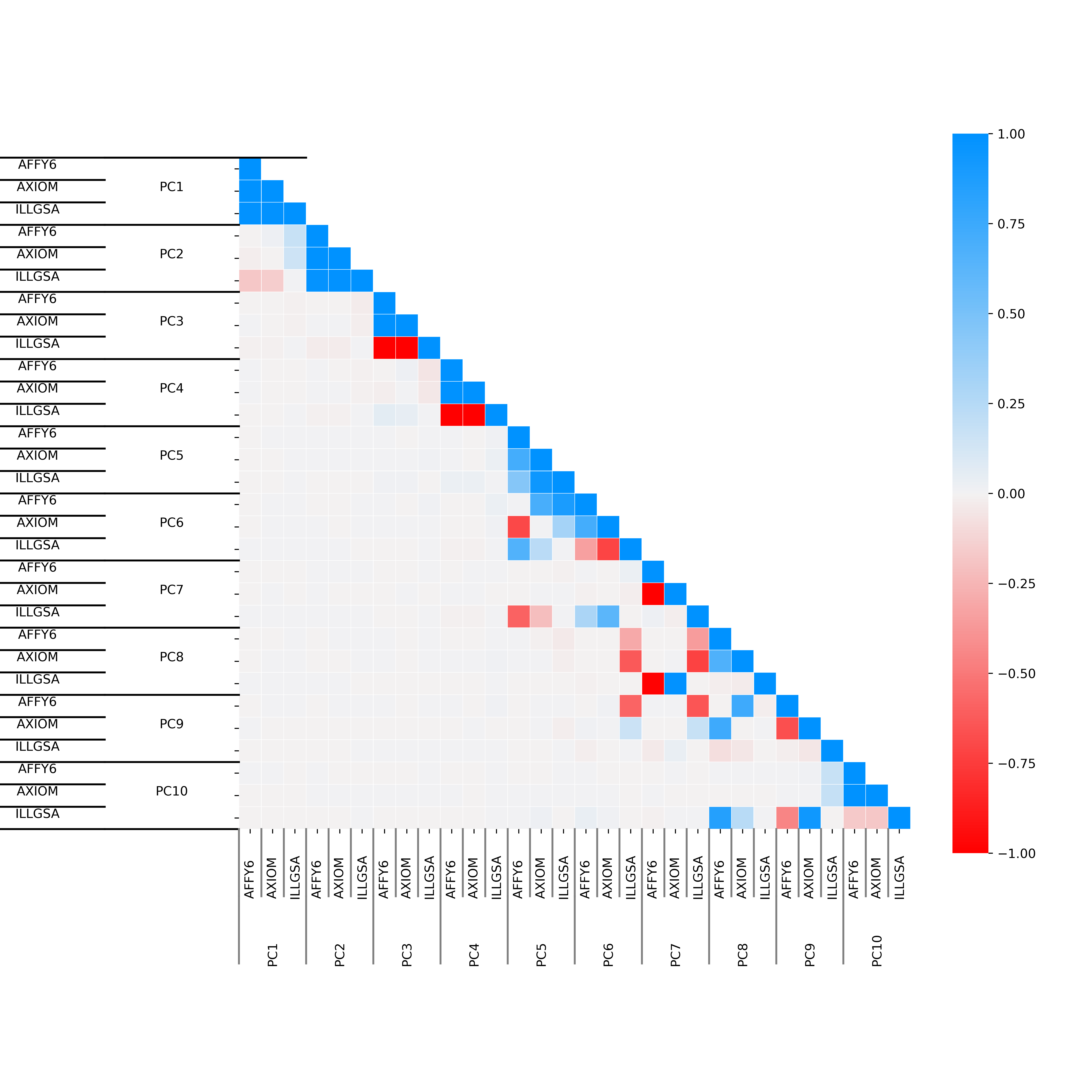

Supplement: Supplementary file 1 [file genes-14-01497-s001.zip › Supplementary_Figures/Figure_S4.png]

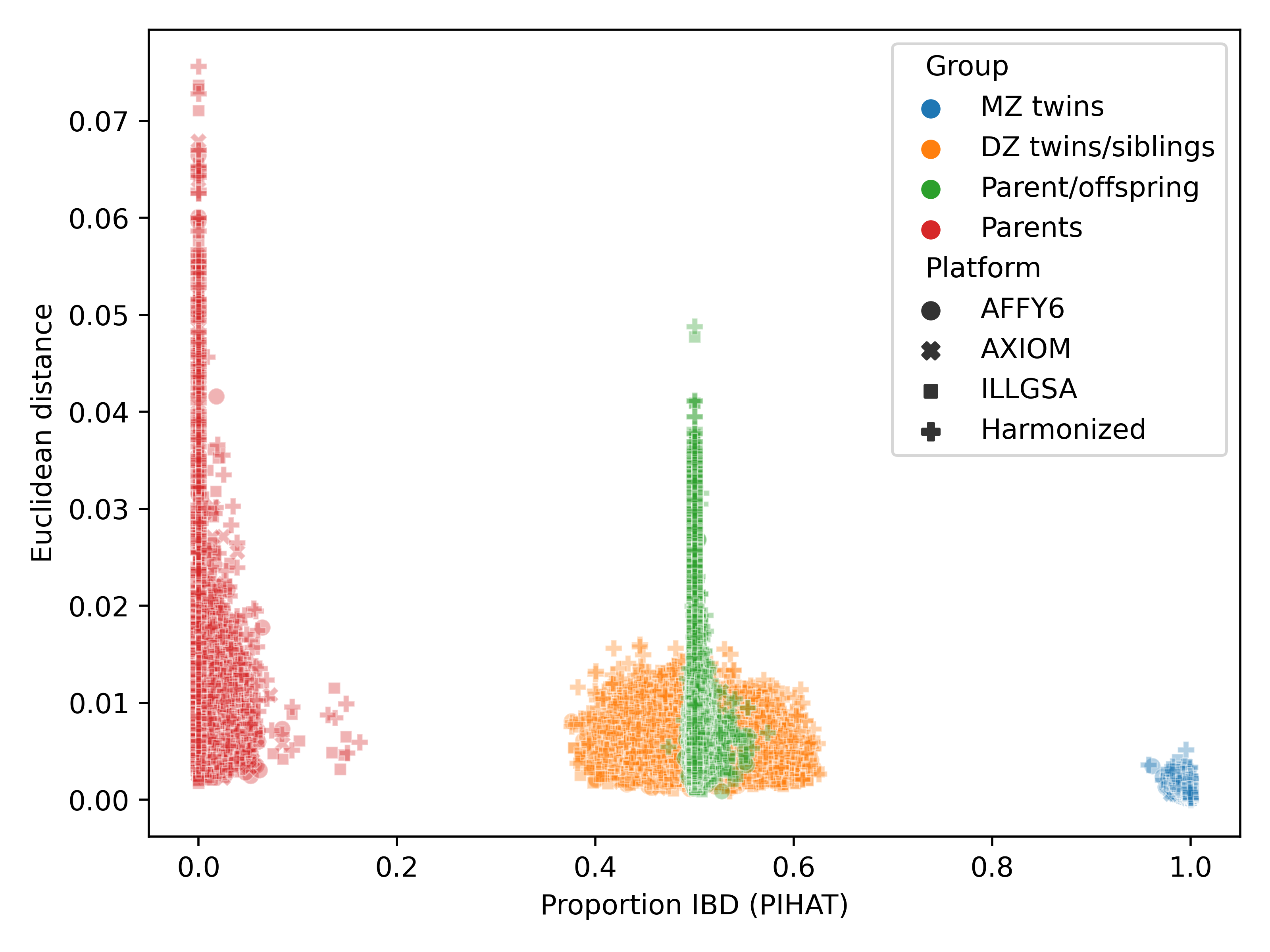

Supplement: Supplementary file 1 [file genes-14-01497-s001.zip › Supplementary_Figures/Figure_S5.png]

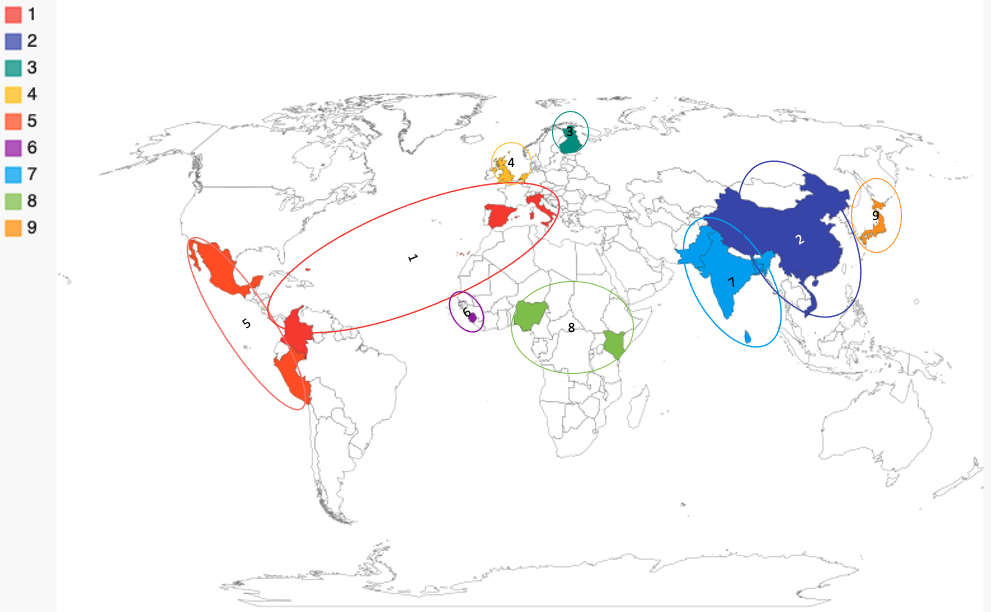

Supplement: Supplementary file 1 [file genes-14-01497-s001.zip › Supplementary_Figures/Figure_S6.png]

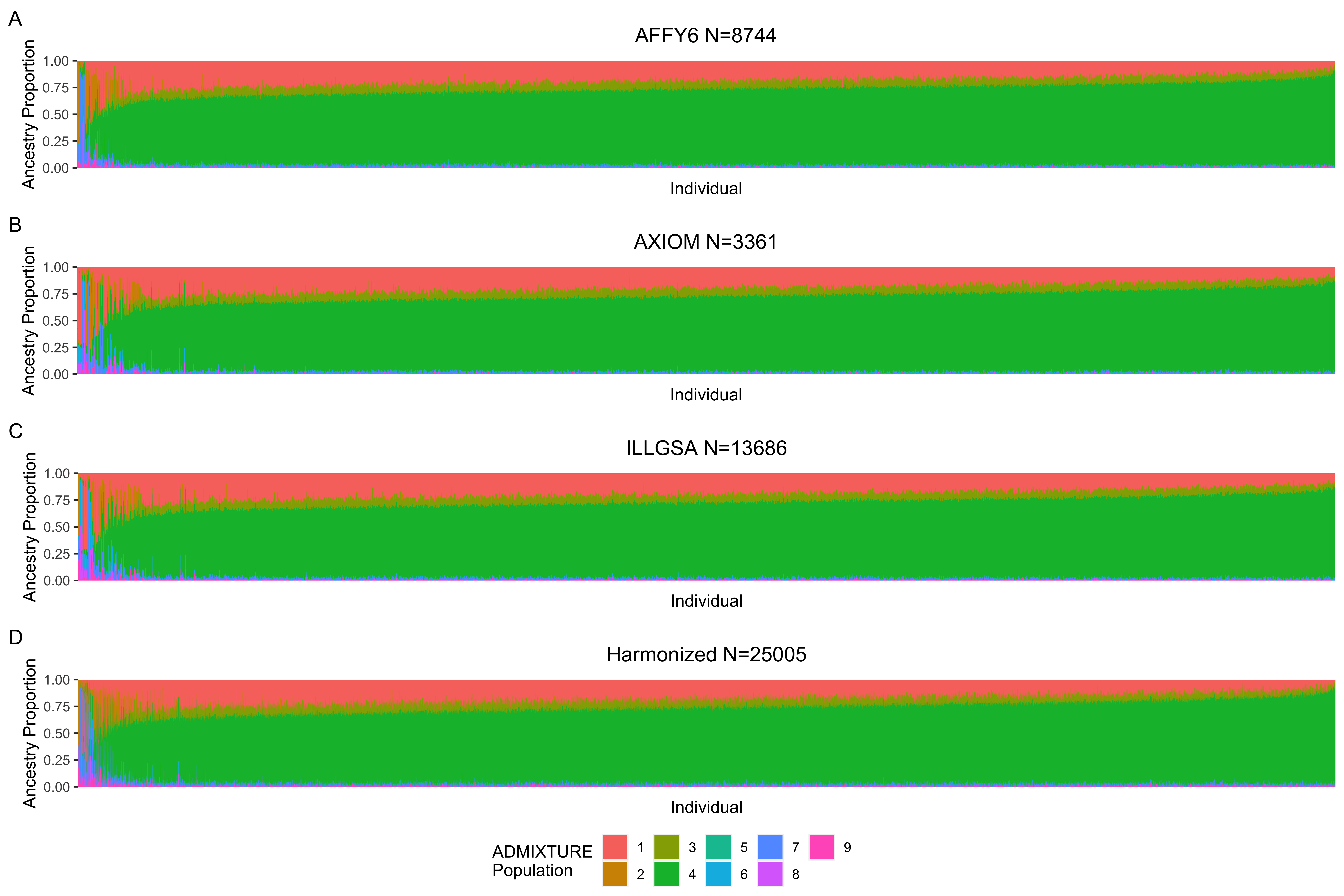

Supplement: Supplementary file 1 [file genes-14-01497-s001.zip › Supplementary_Figures/Figure_S7.png]

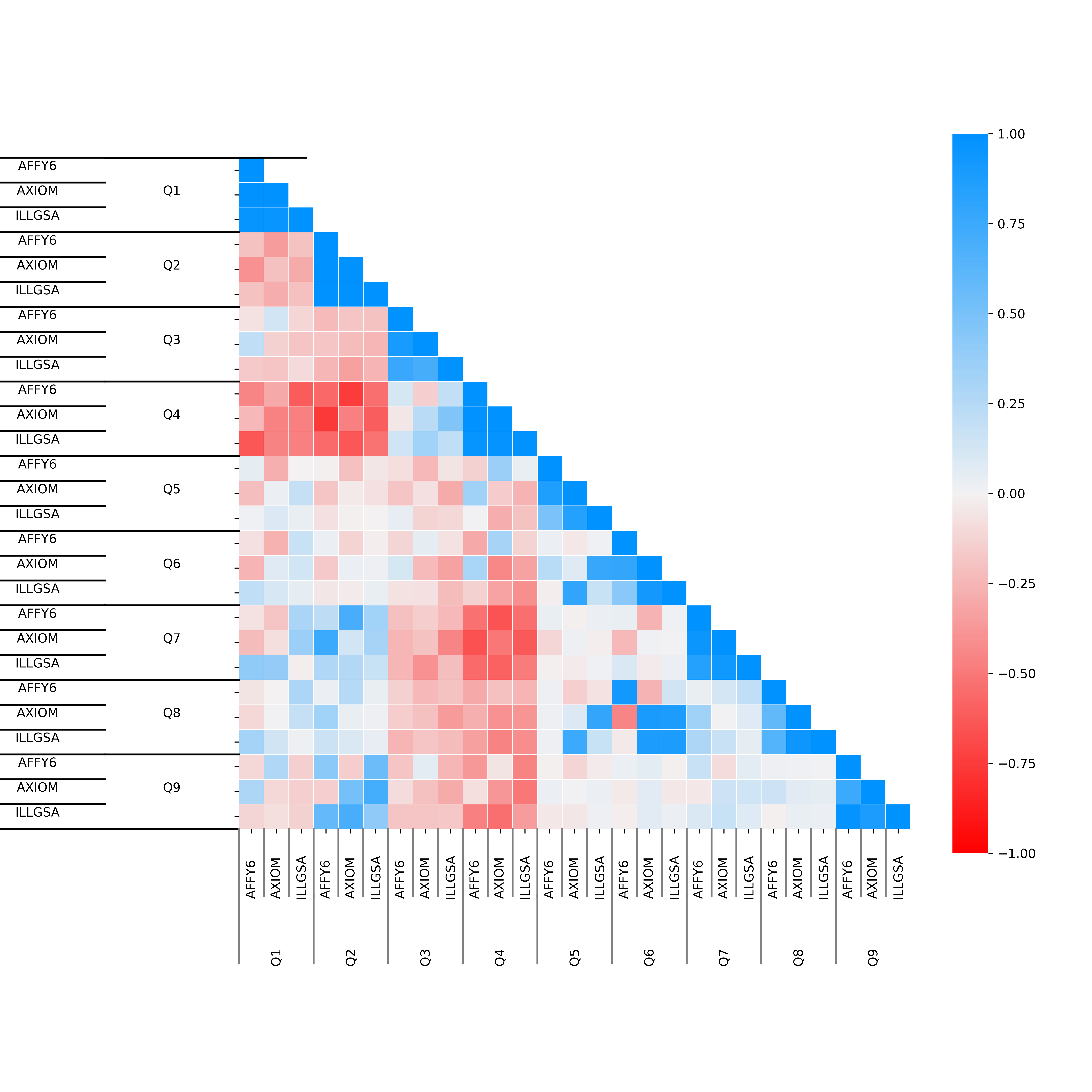

Supplement: Supplementary file 1 [file genes-14-01497-s001.zip › Supplementary_Figures/Figure_S8.png]
